# Diffusion MRI for Assessment of Bone Quality; A Review of Findings in Healthy Aging and Osteoporosis

**DOI:** 10.1002/jmri.26973

**Published:** 2019-11-11

**Authors:** Anahita Fathi Kazerooni, Jose M. Pozo, Eugene Vincent McCloskey, Hamidreza Saligheh Rad, Alejandro F. Frangi

**Affiliations:** ^1^ Department of Radiology, Perelman School of Medicine University of Pennsylvania Philadelphia Pennsylvania USA; ^2^ CISTIB Centre for Computational Imaging & Simulation Technologies in Biomedicine, School of Computing and School of Medicine University of Leeds Leeds UK; ^3^ Department of Oncology & Metabolism, Mellanby Centre for Bone Research, Centre for Integrated research in Musculoskeletal Ageing University of Sheffield Sheffield UK; ^4^ Quantitative MR Imaging and Spectroscopy Group, Research Center for Molecular and Cellular Imaging Tehran University of Medical Sciences Tehran Iran; ^5^ Department of Medical Physics and Biomedical Engineering Tehran University of Medical Sciences Tehran Iran; ^6^ LICAMM Leeds Institute of Cardiovascular and Metabolic Medicine, School of Medicine University of Leeds Leeds UK

**Keywords:** bone quality, diffusion MRI, osteoporosis, aging, bone marrow, trabecular bone

## Abstract

Diffusion MRI (dMRI) is a growing imaging technique with the potential to provide biomarkers of tissue variation, such as cellular density, tissue anisotropy, and microvascular perfusion. However, the role of dMRI in characterizing different aspects of bone quality, especially in aging and osteoporosis, has not yet been fully established, particularly in clinical applications. The reason lies in the complications accompanied with implementation of dMRI in assessment of human bone structure, in terms of acquisition and quantification. Bone is a composite tissue comprising different elements, each contributing to the overall quality and functional competence of bone. As diffusion is a critical biophysical process in biological tissues, early changes of tissue microstructure and function can affect diffusive properties of the tissue. While there are multiple MRI methods to detect variations of individual properties of bone quality due to aging and osteoporosis, dMRI has potential to serve as a superior method for characterizing different aspects of bone quality within the same framework but with higher sensitivity to early alterations. This is mainly because several properties of the tissue including directionality and anisotropy of trabecular bone and cell density can be collected using only dMRI. In this review article, we first describe components of human bone that can be potentially detected by their diffusivity properties and contribute to variations in bone quality during aging and osteoporosis. Then we discuss considerations and challenges of dMRI in bone imaging, current status, and suggestions for development of dMRI in research studies and clinics to segregate different contributing components of bone quality in an integrated acquisition.

**Level of Evidence:** 5

**Technical Efficacy Stage:** 2

J. Magn. Reson. Imaging 2020;51:975–992.

**CME Information: Diffusion MRI For Assessment of Bone Quality; A Review of Findings in Healthy Aging and Osteoporosis**

If you wish to receive credit for this activity, please refer to the website: http://www.wileyhealthlearning.com/JMRI

**Educational Objectives**

Upon completion of this educational activity, participants will be better able to:

- Describe the key factors contributing to variations in diffusion process in bone during healthy aging and osteoporosis
- Describe the potential role of diffusion MRI for assessment of bone quality in healthy aging and osteoporosis

**Activity Disclosures**

No commercial support has been accepted related to the development or publication of this activity.

**Faculty Disclosures:**

**Editor‐in‐Chief:** Mark E. Schweitzer, MD, discloses consultant fees from MCRA and MMI.

**CME Editor:** Mustafa R. Bashir, MD, discloses research support from GE Healthcare, Madrigal Pharmaceuticals, NGM Biopharmaceuticals, Siemens Healthcare and Taiwan J Pharma, and consultant fees from RadMD.

**CME Committee:**

Bonnie Joe, MD, PhD, discloses author royalties from UpToDate.

Tim Leiner, MD, PhD, discloses research grants from Bayer Healthcare and Philips Healthcare

Shreyas Vasanawala, MD, PhD, discloses research support from GE Healthcare, and founder's equity in Arterys.

Eric Chang, MD, Feng Feng, MD, and Bruno Madore, PhD; no conflicts of interest or financial relationships relevant to this article were reported.

**Authors:**

Anahita Fathi Kazerooni, PhD, Jose M Pozo, PhD, Eugene Vincent McCloskey, MD, Hamidreza Saligheh Rad PhD, and Alejandro F. Frangi PhD reported no conflicts of interest or financial relationships relevant to this article.

This activity underwent peer review in line with the standards of editorial integrity and publication ethics. Conflicts of interest have been identified and resolved in accordance with John Wiley and Sons, Inc.'s Policy on Activity Disclosure and Conflict of Interest.

**Accreditation**

John Wiley and Sons, Inc. is accredited by the Accreditation Council for Continuing Medical Education to provide continuing medical education for physicians.

John Wiley and Sons, Inc. designates this journal‐based CME activity for a maximum of 1.0 *AMA PRA Category 1 Credit™*. Physicians should only claim credit commensurate with the extent of their participation in the activity.

For information on applicability and acceptance of continuing medical education credit for this activity, please consult your professional licensing board.

This activity is designed to be completed within 1 hour. To successfully earn credit, participants must complete the activity during the valid credit period, which is up to two years from initial publication. Additionally, up to 3 attempts and a score of 70% or better is needed to pass the post test.

OSTEOPOROSIS, a systemic, metabolic skeletal disorder characterized by reduced bone strength, predisposes patients to an increased risk of fragility fractures, with consequent morbidity and mortality.^1^ Mechanical competence of bone depends on both the applied force and bone strength and tolerance in resisting this force.^2^ Conventionally, bone mineral density (BMD) measurement based on dual energy x‐ray absorptiometry (DXA) is considered the principal "gold standard" for clinical assessment of bone strength and vulnerability to fracture.^3^ Osteoporosis is defined as a condition when BMD falls below the range of –2.5 standard deviation from the mean BMD for the normal young female population.^4^

Nonetheless, the BMD definition of osteoporosis only considers bone quantity, which is not a comprehensive predictor of susceptibility of bone to fracture. Bone quality, as a representative of a wide range of features including bone micro‐ and macrostructure, mineralization, vascularization, and bone marrow composition also contributes to bone strength.^5^, ^6^ In this context, magnetic resonance imaging (MRI) has gained increasing interest as a useful imaging tool for investigation of numerous structural and physiological properties of both bone and bone marrow.^5^ Specifically, diffusion MRI (dMRI) techniques may provide insights about cellularity, homogeneity, directionality, and perfusion variations due to pathophysiological changes to the bone marrow caused by osteoporosis.^7^ Diffusion contrast encoding MRI, or dMRI, has gained broad applications in the diagnosis and monitoring of patients with many diseases, in all body organs, including bone marrow. However, due to limitations in acquisition and lack of appropriate biophysical modeling, its application and beneficial diagnostic value has not yet been established in osteoporosis, with only sporadic experiments having been carried out in the literature.

In this review, we address the value of dMRI in assessment of bone quality in age‐related bone loss and osteoporosis, which can be potentially measured by diffusion imaging. We begin with describing the main components of bone and its structural and physiological aspects related to aging and osteoporosis that can potentially be measured by dMRI, including water, fat, and perfusion. We then review relevant published studies of dMRI in the understanding and assessment of osteoporosis, analyzing their current technical development, difficulties, and potential to become clinically accepted tools.

## Literature search

MEDLINE, EMBASE, and Google Scholar databases were electronically searched to identify relevant studies including the following keywords and subject headings: ("bone") and ("DWI" or "diffusion weighted imaging" or "diffusion weighted MRI" or "DTI" or "diffusion tensor imaging" or "Intra‐voxel Incoherent Motion" or "IVIM" or "perfusion weighted MRI" or "PWI" or "dynamic contrast‐enhanced MRI" or "DCE‐MRI" or "fat fraction" or "magnetic resonance spectroscopy" or "MRS") and ("osteoporosis" or "aging"). No limitations were enforced on the year of publication. Following the initial search, the articles listed in the references of identified studies were scanned. The search included articles available online until March 2019. Exclusion criteria were nonindexed conference papers or abstract‐only publications.

## Bone Microstructure and Its Changes During Aging and Osteoporosis

Mature bone is a complex composite tissue consisting of a partly hematopoietic and partly fatty marrow surrounded by solid bone matrix. The solid organic substrate of bone comprises type‐I collagen (~50%) solidified by mineral calcium hydroxyapatite crystals (~35%) with the remaining volume (~15%) comprising bone water.^6^ The mineral hydroxyapatite crystals add extra rigidity to the collagen fibers.^8^ Bone is a unique connective tissue, as its physiologically‐mineralized matrix undergoes persistent remodeling and regeneration.^9^ Each of the bone tissue components, despite their difference in composition, structure, and function, contributes to the overall bone function.

Structurally, bone tissue consists of trabecular or cancellous bone filled with bone marrow, and cortical or compact bone. Bone marrow is a reservoir of bone and stem cells, and the blood vessels within the marrow play an integral role in blood circulation of the bone. Damage to the bone marrow hinders the functions of bone and periosteum.^10^ Cortical bone is the main constituent of long bones in the extremities, with ~90% bone and 10% pore spaces. Larger pore spaces are mainly related to Haversian canals in the center of the osteon, with smaller pores belonging to the lacuna‐canalicular pores containing osteocytes. The cortical bone organization renders resistance to bending, torsional, and shear forces. Trabecular bone is predominantly present at the ends of long bones around joints and within the axial skeleton with a 3D network of plates and struts immersed in bone marrow and encased with a relatively thin layer of compact bone.^3^ The microarchitecture of trabecular bone delivers tissue resistance against the applied loading forces and contributes to bone strength independently of bone mass.^11^

**Figure 1:**
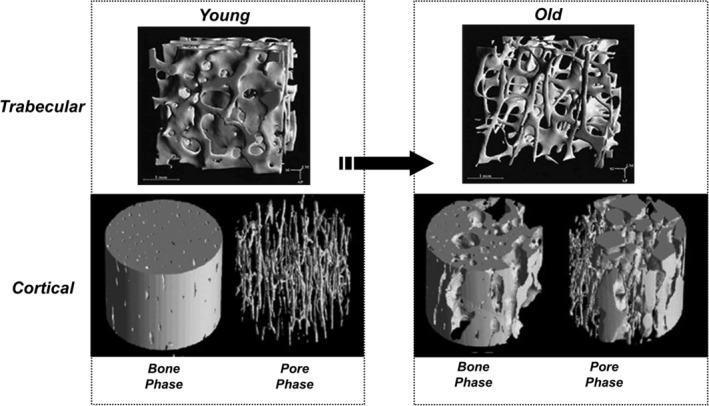
The effect of aging and osteoporosis on bone loss. On the left side, the trabecular and cortical bone images of a young adult, and on the right side, the corresponding images of an old adult are indicated. Porosity increases with advancing age and this process is further accelerated in osteoporosis. This effect is compounded with a loss in mechanical competence. Top row: Reprinted from "Direct three‐dimensional morphometric analysis of human cancellous bone: microstructural data from spine, femur, iliac crest, and calcaneus," Rüegsegger et al, J Bone Min Res 1999;14:1167–1174, with permission from John Wiley & Sons (License No. 4661520359914). Bottom row: Reprinted from "Age‐dependent change in the 3D structure of cortical porosity at the human femoral midshaft," Cooper et al, Bone, 2007;40:957–965, with permission from Elsevier (License No. 4661520195281).

With aging and osteoporosis, bone loss is accompanied with deterioration of microarchitecture of the trabecular bone network, where along with thinning of the rods and plates the topology also changes, with significant conversion of plates to rods, resulting in detachment of trabeculae (Fig. 1). Cortical bone becomes thinner and the porosity increases. Both processes lead to loss of bone strength.

The matrix of bone is constantly being turned over. Osteoclast cells resorb the bone and osteoblast cells form new bone.^12^ Three main mechanisms contribute to pathophysiology of healthy aging and osteoporosis: 1) during healthy aging, the number and function of osteoblasts reduce significantly, leading to decreased bone formation; 2) with age, and particularly in early postmenopausal women, osteoclastic bone resorption is accelerated; and 3) bone marrow fat content increases, affecting osteoblastic differentiation and function, osteoclastic activity, and mineralization.^13^ These processes result in a formation‐resorption imbalance causing progressive bone loss.^12^

Below, we detail these changes in bone structure and function for the different bone components.

### *Bone Water Content*

Bone consists of a solid mineral matrix filled with bone marrow where a total ~15% of volume comprises bone water.^6^ Bone water plays a pivotal role as a mediator for mechanical transduction that confers viscoelasticity to bone, and for transmission of nutrients and waste products.^14^ Two main types of bone water can be distinguished: free and bound water. Extracellular, pore, or free water occupies different porosity levels of bone tissue, while bound water is a structural component of the mineral phase, tightly attached to hydroxyapatite crystals, or loosely bound to the organic phase, ie, collagen type I, noncollagenous proteins, and other components.

**Figure 2:**
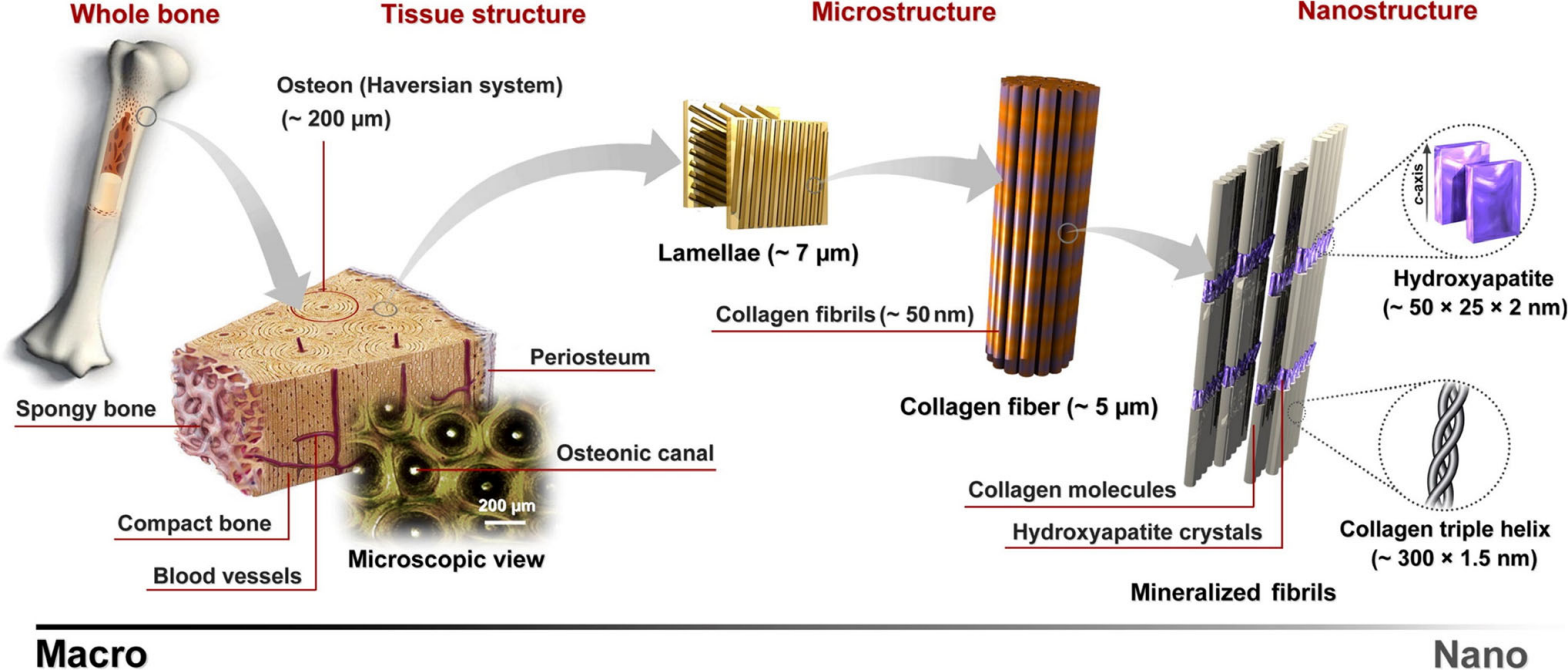
This figure illustrates the porous structure of bone at multiple length scales (from macro to nanoscale). If we magnify a section of cortical bone we see that it consists of osteons which, in turn, are made of lamella with osteocytes interspersed. Each lamella consists of collagen fibers which in turn are made up of fibrils. These are composed of an assembly of collagen molecules with calcium apatite‐like inorganic crystals interspersed as shown here and the basic building block is the collagen triple helix. Reprinted from "Synthesis methods for nanosized hydroxyapatite with diverse structures," Sadat‐Shojai et al, Acta Biomater 2013;9:7591–6721 with permission from Elsevier (License No. 4661520035459).

Bone forms a nested pore architecture, in which the solid and fluid structures have poroelastic interactions with each other. Bone tissue presents three levels of porosity or pore spaces^15^ (Fig. 2) that can be described as a set of nested or hierarchical porosities resembling Russian matryoshki dolls of decreasing sizes placed inside one another. These nested pores are connected in a way that pore water can be interchanged between different pore sizes.^16^ The macroscopic pore size corresponds to the vascular porosity comprised of Haversian and Volkmann's canals (average diameter ~50 μm). The next porosity size class belongs to the lacuna‐canalicular network comprising the volume around osteocytes and their cellular extensions (average diameter ~100 nm). The final level of bone hierarchical porosity, which is considered to have a smaller contribution to fluid flow, corresponds to the spaces within the collagen‐hydroxyapatite structure (average diameter ~5 nm).^17^ The water pool within the two former bone porosity levels is referred to as free water, and the latter is the bound water pool.^18^

Fluid flow between vascular and the lacuna‐canalicular pores accomplishes three critical tasks: 1) it transports nutrients and oxygen to the cells; 2) it removes the waste products of the cells; and 3) it exerts a force (mechano‐transduction role) on the mechano‐sensory osteocyte cells so they adapt the bone mass and structure to the mechanical demands (mechano‐adaptation role).

Bone water flow contributes to transmitting the remodeling signals to bone cells, permitting responses to the applied mechanical loads. Bone strains generate interstitial fluid flow through the lacuna‐canalicular system producing streaming potentials and shear stress. Osteocytes located inside the lacunae (pores) of the lacunar‐canalicular porosity sense the mechanical loading and are activated by the drag induced by the fluid flow.^19^ Abnormally high fluid flow informs the osteocytes to signal osteoblasts for bone formation, while abnormally low flow recruits the osteoclasts to absorb the existing bone.^20^ This bone remodeling is beyond density adjustment, determining also the orientation of trabeculae and osteons along the loading direction based on the strain gradients.^21^

Mechano‐transduction is a fundamental concept underlying the pathophysiological processes involved in bone loss due to lack of mobility or long‐term weightlessness (eg, space flights).^22^ With aging, the imbalance in bone turnover results in decreased bone strength and increased risk of fractures. Furthermore, it has recently been suggested that, with aging, the shape of osteocytes and their lacunae notably alters, leading to changes in the osteocytes mechano‐sensitivity, altering their adaptation to the local mechanical loading.^23^

Segregation of free and bound water is important, as there exists age‐related decrease in bound water^24^ and age‐related increase in porosity (free water).^25^ Studies have demonstrated that bound and free water are more correlated with the ability to withstand fracture than age; bound water contributes to the ability of collagen to tolerate tensile stress, and free water in the pores renders elastic stiffness.^26^

### *Bone Blood Flow and Perfusion*

Principal arteries penetrating the cortex and perfusing the medullary sinusoids are responsible for supplying bone with nutrients; blood leaves the tissue through small veins. Normal bone demands a considerable blood flow (5.5–11% of the cardiac output) to supply the bone and endothelial cells and bone marrow with oxygen and nutrients and excrete carbon dioxide and other waste products.^27^ The rich blood supply of bone allows rapid growth, constant remodeling, and responsiveness to the applied mechanical loadings, as well as metabolic responses, eg, calcium or acid–base balance.^27^ In the skeletal system, osteogenesis and angiogenesis are closely coupled, so it signals the chondrocytes and other bone cells through vascular endothelial growth factor (VEGF) to regulate the formation of vasculature and blood perfusion and has an influential role in the generation of new bone.^28^

Exercise and increased local mechanical strain on bone is associated with bone blood circulation alterations,^29^ while bone loss due to disuse or immobility is associated with decreased bone blood circulation.^27^ Dynamic mechanical loading of the poroelastic bone matrix intensifies the intramedullary pressure, driving interstitial fluid flow within the lacuna‐canalicular porosity and the osteocytic adaptation response is activated.^27^ Conversely, angiogenic factors produced by bone cells cause directional angiogenesis to maintain blood perfusion within the requisite level during the whole remodeling process.^30^ Vascular pressure may not directly elevate transport within lacuno‐canalicular porosity. However, bone blood flow changes could influence this process either through alteration of bone interstitial fluid to stimulate the osteocytes or by changing the milieu of the bone marrow. The latter stimulates bone‐lining cells near the marrow without activating the osteocytes. Both effects induce bone remodeling.^27^

Relationships between aging/osteoporosis and variations of blood flow and perfusion in bone have been stated in several studies. Several risk factors associated with osteoporosis, such as diabetes, postmenopausal status, hypertension, cardiovascular pathologies, and lack of physical activity are also risk factors for vascular diseases.^22^ In postmenopausal women, with deficiency of estrogen, the risk of osteoporosis is elevated, possibly because estrogen directly modulates angiogenesis through endothelial cells^31^ and estrogen reduction increases osteoclastic resorption and reduces bone mineral density^32^ and vertebral blood flow.^33^ Reduction of blood flow during aging is another contributing factor for osteoporosis in postmenopausal women.^34^ This reduction may be attributed to a progressive decline of oxygen consumption and vascular conductance and an increase of vascular resistance during aging.^35^ Increased resistance of blood vessels and decreased bone mineral density during aging and osteoporosis have reciprocal cause and effect: reduced bone perfusion causes decreased intramedullary pressure, which results in higher osteoclastic resorption and lower osteoblastic bone formation. This effect produces an outflow of calcium from the bone into the blood vessels, resulting in mineralization of vessel walls, which further elevates the resistance of capillary walls and reduces perfusion.^27^

Besides changes in blood flow, vessel wall characteristics such as interstitial space and capillary density notably decrease in patients with lower BMD, which may be determinant factors for degraded perfusion function in osteoporotic patients.^36^ Gender‐related variations in perfusion have been reported, suggesting significantly higher marrow perfusion in female subjects than males younger than 50 years, while perfusion significantly decreases in females vs. male subjects older than 50 years.^37^ The amount of perfusion reduction in association with BMD may differ for each anatomical region.^38^

### *Bone Marrow Fat Content*

Bone marrow accounts for ~4–5% of the human total body weight and around 75% of the trabecular or cancellous bone tissue. According to cellular composition and vascularization, two distinct types of bone marrow exist: red marrow, mostly composed of hematopoietic cells, and yellow marrow, mainly containing adipocytes. Osteoblasts and adipocytes are derived from mesenchymal stem cells (MSCs), while osteoclasts are produced from hematopoietic precursors.^13^ Both hematopoietic and mesenchymal cells coexist from embryonic stages throughout adulthood, but the number and function of these cells, including osteoblasts, decline after the second decade of life, while the number and volume of adipocytes increase. Losing estrogen in postmenopausal women also promotes a switch in differentiation of MSCs into adipogenic instead of an osteogenic lineage.^13^ Adipocytes are potentially self‐promoting, initiating differentiation of more adipocyte cells, and are metabolically active, suppressing osteogenesis. Bone marrow fat (BMF) may be partly responsible for bone loss and osteoporosis.^5^

The proportion of fat and nonfat cells in the marrow depends on gender, age, and anatomical location. Higher BMF is expected in females than males, with a sharp increase of BMF in females over an age range of 55–65, in contrast to a steady gradual increase over the lifetime in males.^39^ At birth, bone is predominantly filled by red bone marrow (with nearly no marrow fat), while it becomes progressively substituted by fatty yellow marrow and a balance between red and yellow marrow is reached by the age of 25. Nonetheless, yellow marrow reconversion to red marrow can occur as a result of increased demand for hematopoietic cells.^40^ Red marrow has rich vasculature and plays a key role in producing mature blood cells. In adults, red marrow is mainly confined to the axial skeleton, ribs, and breastbone. In long bones, yellow marrow can be found in the diaphysis and epiphyses, while red marrow is in the metaphysis.^41^ Still, differences in perfusion parameters of red marrow compared to yellow bone marrow can be observed in the femoral head and neck that contain lower amounts of microvasculature.^42^ Therefore, characterizing BMF and perfusion may provide helpful information about bone remodeling disorders.

A close association exists between an increase in BMF and decreased BMD.^43^ In aging and systemic diseases like osteoporosis, BMF is elevated.^44^ A potential dynamic relationship between fatty acids and metabolic demands of the cells has been shown,^45^ implying an association between bone marrow adiposity and metabolism.

## Diffusion MRI in the Study of Bone Aging and Osteoporosis

MRI has several appealing features for measurement of bone quality: it is nonionizing, provides the possibility of direct acquisition of images at arbitrary orientation, and several physiological aspects of bone, such as fat and water content, diffusion, and perfusion of bone marrow can be captured.^5^ This has led to its utilization in characterization of bone marrow in osteoporosis and age‐related bone loss^5^ (Fig. 4).

Conventional and Dixon MRI methods are ideal for measurement of BMF and discrimination of fat from hematopoietic marrow, as these two components appear with different signal intensities on MR images.^46^ Furthermore, proton magnetic resonance spectroscopy (1H‐MRS) and chemical shift encoding‐based water‐fat imaging allow for quantitative measurement of fat fraction in the bone marrow. MRS studies of vertebral marrow fat suggest an increase in BMF with aging^47^ and osteoporosis.^48^

Association of perfusion and blood flow changes with aging and osteoporosis has been documented using dynamic contrast‐enhanced (DCE) MRI studies, showing significant correlation between reduced vertebral bone marrow perfusion indices and BMD.^49^

Measurement of bone water content can be performed by utilizing specifically‐designed MRI methods. The solid phase of the bone has very short relaxation times (T_2_ < 1 msec), so its rapidly decaying nuclear magnetic resonance (NMR) signals are not detectable by conventional MRI methods. Changes to the microstructure of trabecular bone in osteoporosis can be detected indirectly by visualizing signal voids within the hyperintense signal of the bone marrow. With development of new acquisition methods, motion compensation, and postprocessing techniques in MRI, analysis of the 3D meshwork of the trabecular bone in resolutions of 100–200 μm has become possible.^11^

In cortical bone imaging, MRI signal completely decays before activation of the receive mode in conventional clinical MRI scanners. This issue leaves no chance of measuring T_1_, T_2_ (T_2_*), or proton density parameters, leading to signal void in the bone region on conventional MR images. Solid‐state MRI methods based on ultrashort echo time (UTE) or zero echo time (ZTE) imaging with very short TEs^50^, ^51^ have made it possible to recover the very short T_2_* or T_1_ of the bone water and acquire signal directly from the cortical bone.

In bone tissue, dMRI has been used to quantitatively assess pathophysiological changes of bone marrow beyond tissue relaxation parameters and fat content.^52^ dMRI is sensitive to random movement of water molecules within a space resulting from collision of molecules against each other, referred to as *self‐diffusion*, and characterized by a diffusion coefficient *D*_*self*_. In biological tissues, self‐diffusion is restricted by cellular microstructure and, therefore, the measured diffusion coefficient within the tissue is smaller than the diffusion coefficient of free water molecules, and is usually direction‐dependent (anisotropic). The diffusion process is an indispensable physical phenomenon essential for functioning of living tissues.^53^ Therefore, any changes in normal functional properties of the tissues resulting from pathology could be detected early due to alterations in the diffusion process. dMRI acquired in vivo provides information about size, orientation, and shape of tissue microstructure and is sensitive to the pathological processes associated with changes in cellular density and orientation, microvasculature, and permeability of cellular membrane.^54^

Bone undergoes structural and physiological alterations during aging and osteoporosis, weakening bone quality, so BMF content increases, marrow perfusion decreases, bone marrow cellular density decreases, the microarchitecture of trabecular bone deteriorates, affecting its thickness, quantity, and directional properties, and cortical bone water content (diffusive transport of fluid flow) changes. As these alterations influence the diffusion process in the bone tissue, dMRI is a potent tool to characterize bone quality in aging and osteoporosis. dMRI, if properly designed and quantified, has the potential to serve as a tool for providing prospects about directionality of bone microarchitecture and bone marrow cell density.^53^ Unlike qualitative conventional and Dixon MRI, dMRI proffers quantitative metrics; compared to MRS, it has better spatial resolution and, therefore, higher signal‐to‐noise ratio (SNR) and a rapid acquisition; as opposed to DCE‐MRI, it does not require injection of contrast agent for creating a contrast for detecting the physiological changes within the tissue. dMRI could potentially provide a direct and functional metric of displacement of water molecules through diffusive transport of cortical bone fluid flow, in contrast to UTE‐MRI with indirect measurement of bone water content. Finally, through acquisition of diffusion in internal magnetic field gradients, structural visualization of trabecular bone microarchitecture is attainable.

**Figure 3:**
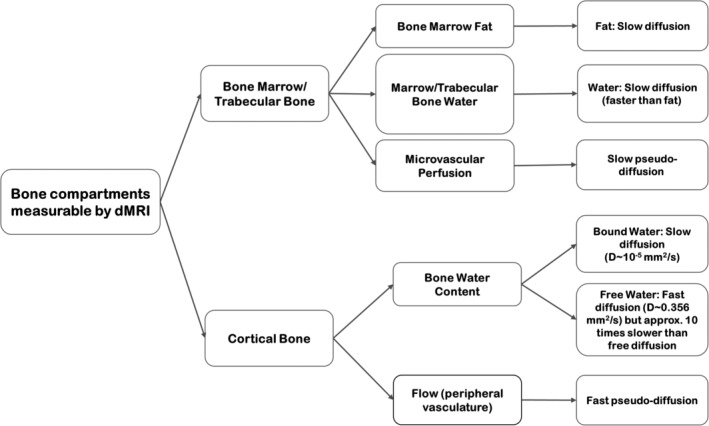
A summary of bone components that undergo variations during aging and osteoporosis and their diffusion properties.

The roles of various MRI methods for characterization of cortical and trabecular bone, and bone marrow, have been described in several review articles (quantitative MRI(/S) of bone marrow^49^, ^52^, ^55^; cortical and/or trabecular bone imaging).^3^, ^6^, ^11^, ^26^, ^56^ Table 1 provides various characteristics of bone during aging and osteoporosis that can be identified by different MRI methods. However, due to insufficient attention to the potential of dMRI for assessment of bone quality in the literature, we will dedicate our focus on this technique. A summary of the bone compartments measurable by dMRI is illustrated in Fig. 3.

**Table 1:** Changes of Bone Properties During Aging and Osteoporosis Based on Imaging Studies

Parameters	Changes due to aging and osteoporosis	Studies
Cortical bone water measured by solid state MRI		
Cortical bone water: free	↑	Review papers 11, 26
Cortical bone water: bound	↓	
Cortical bone blood circulation		
Blood flow	↓	Review papers 107, 108
Bone Marrow Microvascular Perfusion Measured by DCE‐MRI or DCE‐CT		
Blood flow, blood volume, permeability	↓	Ou‐Yang et al 109, 110; Ma et al 36, 111, 112; Dyke et al 37
Maximum enhancement, enhancement slope	↓	Griffith et al 33, 34, 64, 113, 114, 115; Chen et al 116, 117; Biffar et al 55, 118; Wang et al 38, 119;
Time to peak, mean transit time	↑	
Bone marrow fat content measured by 1H‐MRS		
Fat Fraction	↑	Review papers 46, 49
Bone marrow properties (cellularity, anisotropy, microvascular perfusion) measured by dMRI methods		
ADC (DWI)	↓	Review papers 49, 52
MD (DTI)	↓	Manenti et al 72, 73
FA (DTI)	↓	Manenti et al 72, 73
D (IVIM)	↓	Ohno et al 101
D* (IVIM)	N/A	—
f (IVIM)	↓	Ohno et al 101

**Figure 4:**
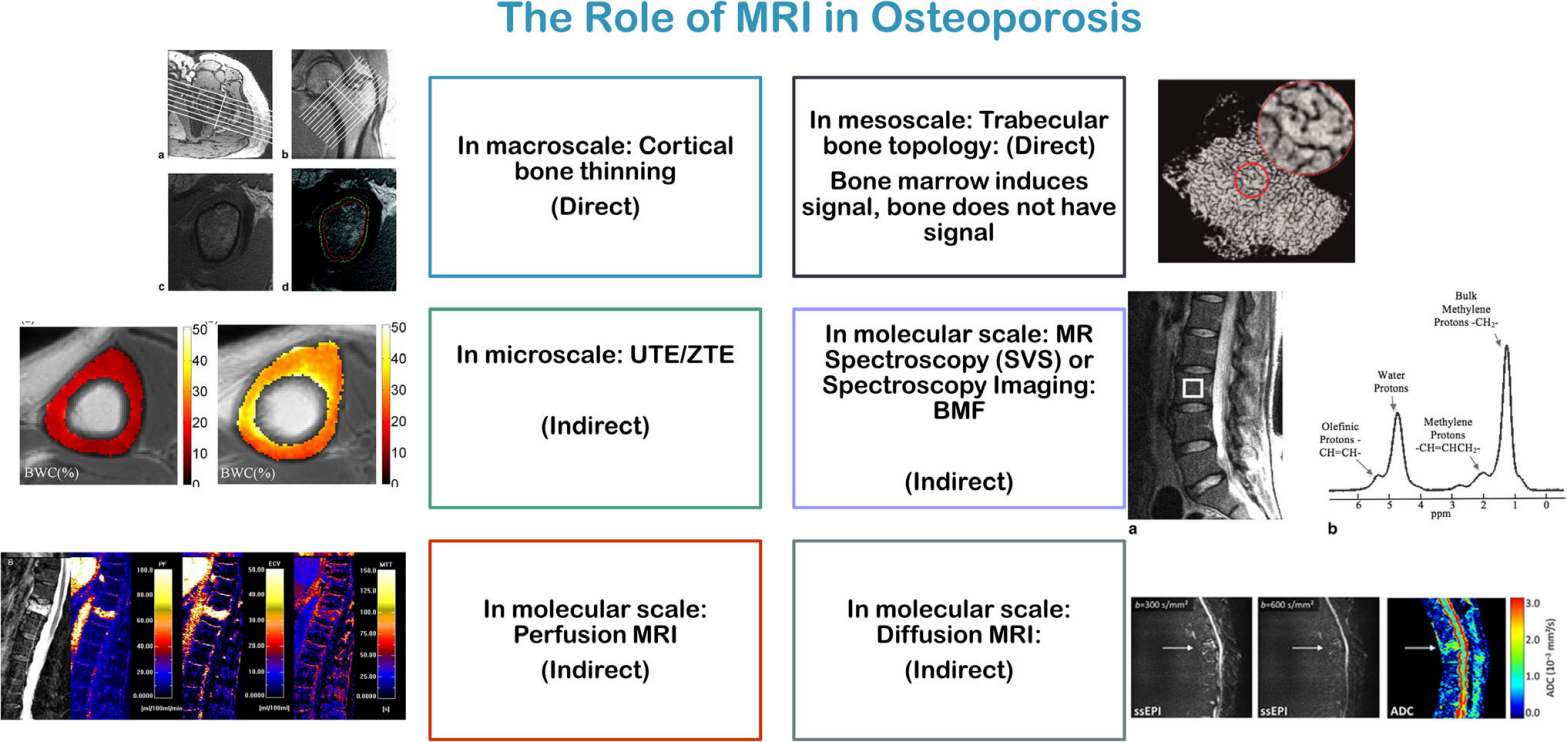
The role of MRI methods in assessment of bone quality in osteoporosis Left: Top row: Reprinted from "Structural and functional assessment of trabecular and cortical bone by micro magnetic resonance imaging," Wehrli et al, J Magn Reson Imaging 2007;25:390–409, with permission from John Wiley & Sons (License No. 4675950978985). Left: Middle row: Reprinted from "Quantifying cortical bone water in vivo by three‐dimensional ultrashort echo‐time MRI," Saligheh Rad et al, NMR Biomed 2011;24:855–864, with permission from John Wiley & Sons (License No. 4675960037019). Left: Bottom row: Reprinted from "Diffusion and perfusion imaging of bone marrow," Biffar et al, Eur J Radiol 2010;76:323–328, with permission from Elsevier (License No. 4675961047317). Right: Top row: Reprinted from "Quantitative MRI for the assessment of bone structure and function," Wehrli et al, NMR Biomed 2006;19:731–764, with permission from John Wiley & Sons (License No. 4675970418715). Right: Middle row: Reprinted from "Quantification of vertebral bone marrow fat content using 3 Tesla MR spectroscopy: Reproducibility, vertebral variation, and applications in osteoporosis," Li X et al, J Magn Reson Imaging 2011;33:855–864, with permission from John Wiley & Sons (License No. 4675990169235). Right: Bottom row: Reprinted from "Diffusion imaging of the vertebral bone marrow," Dietrich et al, NMR Biomed 2015;30:e3333, with permission from John Wiley & Sons (License No. 4661511301476).

**Figure 5:**
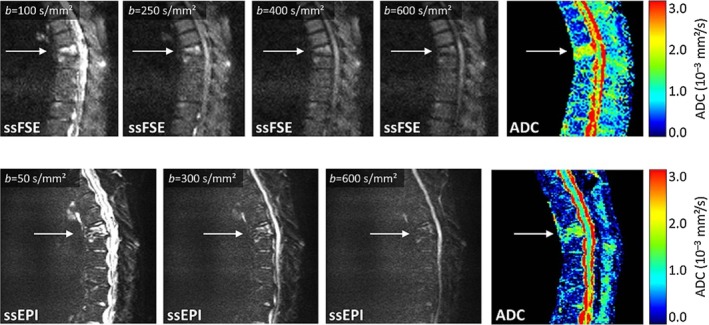
Comparison of dMRI acquisition with ssFSE pulse sequence (top row) and ssEPI (bottom row) at 1.5T: A 69‐year‐old female patient with osteoporotic vertebral compression fracture of T7 has been imaged. At low b‐values, the fracture is hyperintense and at high b‐values it is almost isointense. Compared to the adjacent normal‐appearing bone marrow, the ADC value of the fractured vertebra is significantly higher. Using the ssEPI technique, a notable geometric distortion of the spinal canal can be observed. Reprinted from "Diffusion imaging of the vertebral bone marrow," Dietrich et al, NMR Biomed 2015;30:e3333, with permission from John Wiley & Sons (License No. 4661511301476).

### *(Isotropic) Diffusion‐Weighted Imaging (DWI)*

DWI serves as a tool to characterize the distribution of displacements of the water molecules diffusing (with diffusion coefficient of *D*) in an environment with certain diffusivity properties (viscosity, barriers, etc.) and over a particular observation duration (Δ). In biological tissues, the presence of hindrances, such as membranes and macromolecules, obstruct the free random walk of the water molecules. DWI in a given measurement duration detects the apparent diffusion coefficient (ADC or *D*_*app*_) of the tissue, which is less than *D*_*self*_.

The simplest form of DWI is measurement of ADC in a given gradient direction, assuming the diffusion occurs with no preferential direction, ie, to be isotropic. However, since ADC depends generally on the direction of diffusion encoding, in clinical applications DWI is acquired in three orthogonal measurements and averaged to obtain a better estimation of ADC.

dMRI has found broad interest in the investigation of bone marrow. Isotropic DWI has been tested for its capability in revealing the changes of trabecular bone and bone marrow during aging and osteoporosis. Due to thinning of the trabecular bone matrix, the pores become wider and more connected; fat content within the bone marrow increases and blood perfusion decreases. Through aging and osteoporosis, the proportion of water to fat content in bone marrow changes and these alterations are location‐dependent, eg, the water/fat ratio in osteoporotic bone marrow of vertebra differs from femoral neck or calcaneus. The diffusion coefficient of fat (the main constituent of yellow marrow) is 2–3 orders of magnitude lower than water (the main constituent of red marrow) and, accordingly, the diffusion signal is reduced by the presence of fat.

Site‐, gender‐, and age‐related variations in diffusion coefficients have been identified in consistency with dependence of BMF accumulation to the same factors. Different ADC values have been reported in different anatomical sites, including bone marrow,^57^ iliac marrow,^58^ femoral neck,^59^ skull,^60^ and calcaneus,^61^ with decreasing values from vertebral bodies throughout the upper to bottom spine towards femur and calcaneus. Negative correlation has been observed between ADC values and incrementing age and fat fraction in yellow bone marrow,^57^ and positive correlation between ADC and red bone marrow (with higher cellularity and less fat than yellow marrow).^62^ Furthermore, significantly higher ADC values have been reported in female healthy subjects in comparison with healthy male subjects.^63^

During osteoporosis, two counteracting phenomena take place: marrow fat content increases, resulting in reduced ADC, while deterioration of the solid matrix of trabecular bone causes pore enlargement and connection of the neighboring pores, which results in increased ADC. As calcaneus is predominantly occupied by marrow fat (>90%), the latter effect of pore enlargement and increased ADC is dominant.^7^ Decreased ADC values have been documented in subjects with reduced BMD, attributed to accumulation of fat in bone marrow.^63^ Despite a significant increase in the marrow fat content as a function of decreasing BMD, the ADC parameter has not shown statistically significant differences among the osteoporotic, osteopenic, and healthy subject groups.^64^

For a more detailed description of the available studies investigating ADC values within bone marrow in aging and osteoporosis, we refer interested readers to the excellent review articles.^49^, ^52^

Due to inherent limitations existing in the MRI process, such as the achievable spatial resolution, susceptibility difference between cortical bone matrix and soft tissue, and slow diffusivity of water, dMRI in cortical bone has not yet been of research interest and dMRI in bone has been concentrated around investigating the properties of trabecular bone filled with bone marrow. The only study interrogating diffusive transport mechanisms of bone water within cortical pores was carried out by Fernandez‐Seara et al in 2002^65^ on cortical bone specimens from the midshaft of rabbit tibiae immersed in deuterium oxide. In that study, it was demonstrated that the diffusion coefficient of bound water is two orders of magnitude slower than that of the free water pool (*D*_*a*_ ≈ 7.8 × 10^‐5^ in bone vs. *D*_*a*_ ≈ 3 × 10^‐3^ mm^2^/s for free diffusion), so a water molecule within the cortical bone matrix takes 1.24 minutes to travel 100 μm. Some have suggested that the diffusive water transport should be faster in vivo than ex vivo, as the bone water content is higher. However, the aim of this study was not to provide guidelines for dMRI studies, and the results were only pursued for distinction of water pools within the cortical bone for solid‐state MRI studies.

### *Diffusion Tensor Imaging (DTI)*

In anisotropic tissues like bone, where the displacement of water molecules is directional, the scalar ADC cannot completely and accurately describe diffusion.^66^ The expected anisotropic diffusion can be better represented by the diffusion tensor, $\bar{D}$. This tensor can be calculated using DTI, which requires measurements over at least six different directions.^67^ DTI is a versatile tool for measurement of magnitude and direction of proton diffusion in anisotropic and heterogeneous systems. Bone alters its mass and structure in response to physiological demands and the applied mechanical loads resulting in oriented trabeculae and osteons within a heterogeneous and porous bone system. In particular, trabecular bone can be regarded as a porous medium composed of interconnected cavities interspersed with liquid bone marrow,^68^ which exhibits directional anisotropy of the architecture.

The principal diffusivities (eigenvalues) of the diffusion tensor correspond to the diffusion along the principal directions (eigenvectors) parallel and perpendicular to the tissue fibers, the tensor trace provides the mean diffusivity (MD), and the variance of the three eigenvalues about their mean defines the fractional anisotropy (FA).^69^, ^70^

Based on preliminary ex vivo studies,^71^ it was suggested that DTI‐derived parameters, MD and FA, have potential in specifying the porous architecture of trabecular bone microstructure in such a way that highly isotropic diffusion (low FA) could be observed in fat, whereas spongy bone regions showed increased variability in size and orientation of trabecular bone.^68^ However, it was observed that by increasing the diffusion time, the contrast of MD and FA between isotropic and anisotropic tissue components decreased, which was attributed to the presence of internal gradients induced by magnetic susceptibility contrast between fat and trabecular bone.^68^

In clinical investigations, DTI has been carried out using a spin‐echo segmented echo planar imaging (EPI) technique along with fat‐suppression to reduce the confounding impact of fat on measurements of MD. Satisfactory reproducibility and statistically significant differences have been found for both MD and FA values for discrimination of osteoporotic and osteopenic from healthy subjects. Both MD and FA have shown significant correlation with fat fraction (FF), calculated using MRS. The combination of MD/FF and FA/FF parameters derived from DTI and MRS was shown to be a potential biomarker for the diagnosis of osteoporosis.^72^, ^73^ Exploitation of fat‐suppressed DTI combined with 1H‐MRS techniques suggests that through appropriate modeling of the trabecular bone compartments, including coexisting fat, trabecular bone, and bone marrow in DTI, acquisition of MRS could be avoided.

### *Decay of Diffusion in Internal Fields (DDIF)*

Cancellous or trabecular bone can be considered a porous system, composed of a solid matrix with holes and cavities, which are filled by bone marrow as a soft tissue. In such a porous network, fat is concentrated in the center of each pore and water along with nonfat components of bone marrow being predominantly present in the boundaries.^7^ Bone minerals (calcium and phosphorus) have higher atomic number and density than those of bone marrow; therefore, the solid phase of the trabecular bone that is interspersed with bone marrow is diamagnetic. Due to a large susceptibility mismatch between the two compartments of trabecular bone, ie, solid trabeculae and liquid bone marrow, when placed in a static magnetic field, internal magnetic field gradients (IMFG) are induced in the interface between these two compartments. IMFG induced in the 3D network of trabecular bone was shown to generate a distribution dependent on the orientation of the static magnetic field with respect to the structure, which is indicative of the trabecular bone network anisotropy and heterogeneity.^56^, ^74^ Based on this concept, IMFG measurements have been used in vivo to provide information about the architecture of rods and plates within the complex meshwork of trabecular bone.^75^, ^76^, ^77^, ^78^

However, the notion of a static dephasing regime does not hold in the bone–bone marrow boundary. Rather, in local magnetic field gradients, diffusion of water protons occurs between the peripheral protons of the bone marrow in each pore and their confining walls, which results in rapid loss of coherence. In other words, protons sense numerous local gradients when diffusing near the interface between trabeculae and marrow. This phenomenon amounts to a diffusion decay of internal fields (DDIF) and, if not accounted for, can produce undesired artifacts. However, it can also be exploited as a measurement method for probing heterogeneous materials. Mainly applied to rocks,^79^, ^80^ this was later extended to trabecular bone.^81^, ^82^ The basic idea is to use the susceptibility‐induced gradients to encode diffusion weighting of the spins near the surface of trabecular bone, especially trabecular surfaces oriented perpendicular to the applied field.^82^

Initial attempts in optimizing DDIF for trabecular bone imaging were performed ex vivo after eliminating bone marrow from the specimens.^81^, ^82^ The challenge in translating the technique to in vivo experiments lies in the diffusion properties of the compartments of bone marrow. As underscored before, the proportion of red to yellow marrow components depends on the anatomical site and age of the individuals under study. As attenuation of diffusion signal in fat is higher than water, yellow marrow may not be very sensitive to internal gradients.^56^ Monte‐Carlo simulations of DDIF and in vivo experiments on healthy volunteers indicated that with increasing marrow fat, DDIF decay time reduces and, therefore, it was suggested that incorporation of marrow fat percentage with DDIF quantification could allow for the diagnosis of osteoporosis.^83^ Nonetheless, recent clinical experiments are suggestive of the feasibility of DDIF measurements even in locations with a predominantly fatty component of the marrow.^84^, ^85^

Recently, based on the idea of susceptibility‐induced magnetic field gradients, a new gradient‐based spin‐echo sequence, which exploits diffusion tensor to discern morphological orientations in the nm–mm range, has been devised and tested on a phantom.^86^ This study motivates future endeavors in extending susceptibility tensor imaging method to highly oriented structures such as trabecular bone.

### *Intravoxel Incoherent Motion (IVIM) Imaging*

Diffusion and perfusion have different biophysical definitions, with different origins and spatiotemporal behavior. Nonetheless, blood water molecules in the arbitrary structure of the capillary network follow a complex motion, which mimics a random walk similar to a diffusion effect.^87^

IVIM is a diffusion MRI method accounting for the impact of both diffusion and perfusion components on diffusion signal^88^; the latter usually referred to as pseudo‐diffusion.^67^, ^89^, ^90^ IVIM is a term referring to the microscopic translational movements of water diffusion and blood microcirculation in the capillary bed in each image voxel.^90^ The idea of IVIM was proposed by Le Bihan^89^ as a method for segregation of incoherent and random motion of tissue protons from that of blood protons (with assumption of negligible exchange between blood and tissue), using a biexponential signal decay equation^91^:

$$S/S_{0}={\mathrm{fe}}^{-\mathrm{bD}*}+\left(1+f\right)e^{-\mathrm{bD}}$$

with *f* as the perfusion fraction, D* as the pseudodiffusion coefficient, *D* as the water diffusion coefficient within the tissue. Reliable estimation of IVIM parameters is dependent on acquisition of DWI in multiple b‐values. In small b‐values, both diffusion and blood flow have confounding effects on ADC measurements (*ADC* ~ *D* + *f/b*), while in higher b‐values approximately over 250 s/mm^2^, ADC is affected almost entirely by diffusion (*ADC* ~ *D*).^91^ The pseudodiffusion coefficient (D*) is sufficiently close to *D* to be captured by MRI in a single acquisition, but significantly different (~10 times faster) to allow convenient separation of both effects by multiple b‐value DWI.

Mechano‐transduction and adaptation mechanisms in bone, induced by a diffusion transport mechanism, occur because of the coupled function of bone blood flow and bone/bone marrow water. Thus, both components are important to explore this mechanism.

Multi‐b‐value imaging has been applied in several studies on bone marrow MRI, mainly with the aim of exploring the optimum b‐value for achieving the highest SNR in the bone marrow compartment.^64^, ^68^, ^71^ Nonetheless, there have been a few attempts in implementing IVIM imaging for measuring perfusion and diffusion in bone and, specifically, in the context of osteoporosis.^52^ Only a few studies have explored IVIM in bone marrow pathologies,^92^, ^93^, ^94^, ^95^, ^96^, ^97^, ^98^, ^99^, ^100^, ^101^ among which only two studies have investigated the relationship between BMD and IVIM‐derived parameters.^93^, ^101^ These studies suggest that with increasing BMD, perfusion‐related (fast) diffusion coefficient, D*, increases and true (slow) diffusion coefficient, *D*, decreases.^93^, ^101^

Biexponential modeling can also account for the separation of other effects. For instance, it has been applied in a few studies to separate the diffusion within fat and water marrow components.^102^, ^103^

A summary of findings and methods employed for IVIM imaging in bone marrow (in all currently available applications) is presented in Table 2 to help the readers in understanding the available techniques and potential of IVIM‐MRI in bone marrow. The reported parameters in these studies are mainly *D*, D*, and *f*, but those reporting FF are also mentioned. Where available, the ADC value (computed based on monoexponential fitting) and FF measured using MRS is reported. As only a limited number of studies have investigated multi‐b‐value dMRI in bone marrow, to get an idea of the range of values for parameters within bone marrow, studies of bone lesions other than osteoporosis are included in this table.

**Table 2:** IVIM MRI Studies in Bone Marrow: Normal or Pathological Cases

				*FF*	*ADC* †	*D*	*f*	D*
				*(%)*	(×10^‐3^ *mm* ^*2*^ */s)*	(×10^‐3^ *mm* ^*2*^ */s)*	(%)	(×10^‐3^ *mm* ^*2*^ */s)*
	Study	Sequence	Study population	(Mean ± STD)	(Mean ± STD)	(Mean ± STD)	(Mean ± STD)	(Mean ± STD)
1	Yeung et al, 2004 93	FS^a^ SE^b^ EPI^c^	Osteoporosis (postmenopausal female)	N/A^d^	0.43 ± 0.09	0.42 ± 0.12	N/A	N/A
			Normal controls (premenopausal female)	N/A	0.49 ± 0.07	0.50 ± 0.09	N/A	N/A
2	Biffar et al, 2011 102	ssFP e	Normal‐appearing vertebral bone marrow in patients with osteoporotic lesions	52 ± 13 ^f^	N/A	0.58 ± 0.14	N/A	N/A
3	Marchand et al, 2014 94	FS EPI	Healthy premenopausal female	N/A	N/A	0.60 ± 0.09	14 ± 6	28 ± 9
4	Ohno et al, 2015 101	SE ssEPI^g^	Healthy male and female subjects	~ 60^h^	~ 0.3 i	~ 0.25	~ 10	~ 5
5	Bourillon et al, 2015 95	SE ssEPI	Multiple myeloma	N/A	0.62 ± 0.17	0.52 ± 0.18	9.42 ± 3.96	25.79 ± 19.31
6	Baik et al, 2017 96	SE ssEPI	Focal marrow abnormalities 1 Benign	N/A	0.44	0.41	9.5	0.24
			2 Malignant	N/A	0.95	0.87	11.2	0.26
7	Dieckmeyer et al, 2017 103	FS SE ssEPI	Healthy male and female subjects	33 ± 12 f	0.30 ± 0.07	0.41 ± 0.09	N/A	N/A
8	Lee et al, 2017 97	FS SENSE	Pelvic bone marrow in patients with cervical cancer	N/A	0.31 ± 0.08	0.29 ± 0.05	44 ± 4	N/A
9	Niu et al, 2017 98	FS SE ssEPI	Acute myeloid leukemia 1 complete remission	N/A	0.49 ± 0.17	0.24 ± 0.04	22.38 ± 5.19	67.22 ± 7.07
			2 Nonremission	N/A	0.48 ± 0.09	0.20 ± 0.03	27.89 ± 8.25	66.80 ± 6.76
10	Park et al, 2017 99	FS ssEPI	Focal vertebral bone marrow lesions	N/A	0.69	0.3	12.5	11.0
11	Yoon et al, 2017 100	FS SE ssEPI	Hepatocellular carcinoma	N/A	0.40	0.36	10.96	24.01

† ADC refers to the diffusion coefficient calculated using mono‐exponential decay model;

a FS = fat saturated;

b SE = spin echo;

c EPI = echo planar imaging;

d N/A = not available;

e ssFP = steady‐state free‐precession;

f FF has been calculated based on Dixon acquisition method;

g ssEPI = single‐shot EPI;

h FF has been estimated using 1H‐MRS examination;

i In this study, the values have not been reported explicitly, the values in this table are approximated from the figures within that paper.

### *Technical Considerations of Diffusion MRI in Bone Imaging*

Although dMRI has become an indispensable imaging technique in clinical diagnosis of a variety of pathologies, in bone imaging it is accompanied with technical complications that need to be considered when designing a proper bone study using dMRI. The technical considerations for optimizing dMRI in bone/bone marrow imaging encompass both aspects of pulse sequences and signal modeling. These two factors are dependent, as signal modeling relies on the choice of pulse sequence and parameter adjustments.

Numerous studies have reported the ADC values of normal (appearing) vertebral bone marrow to fall within the range of 0.2–0.6 (×10^‐3^ mm^2^/s),^52^ which is relatively lower than other body tissues. The wide variability in the reported ADC values in different studies is related to failing to consider different compartments of bone marrow that simultaneously contribute to diffusion signal, and differences in the choice of protocols, including pulse sequences with or without fat‐suppression and b‐values.

dMRI can be carried out by applying diffusion gradients to numerous pulse sequences, the details of which have been addressed elsewhere.^52^ The application of each pulse sequence in studying bone could be restricted by different artifacts, including involuntary motion, eddy currents, and internal magnetic field gradients.^7^

For assessment of diffusion in bone, initially, spin‐echo or stimulated‐echo pulse sequences were upgraded through applying pulse gradients to form pulse gradient spin‐echo (PGSE) or stimulated‐echo (PGSTE) sequences. These sequences provide high SNR and show robustness to inhomogeneity of the magnetic field. However, their acquisition is lengthy and sensitive to motion artifact, which makes them unfeasible for implementation in clinical settings.^7^, ^52^ These sequences were modified by applying line scan diffusion imaging (LSDI) to scan lines as a substitute for the 2D plane, which is less prone to motion and susceptibility artifacts.^52^

Nowadays, motion artifacts in diffusion imaging of bone have been widely avoided by the single‐shot echo‐planar imaging (ssEPI) method, as it provides faster scan time. Yet this method suffers from limited spatial resolution (usually with a matrix size of 128 × 128 pixels^52^) due to fast decay of the T_2_* signal, and sensitivity to inhomogeneity and eddy currents, especially susceptibility artifact induced in the interface of bone and bone marrow.^7^, ^52^ The off‐resonance effect caused by differences in magnetic susceptibility of bone and bone marrow results in geometric distortions, which is a contributing factor for limited spatial resolution in ssEPI.^49^ During recent years, with the advent of advanced gradient hardware, parallel imaging, dynamic shimming, and reduced field of view (rFOV) imaging through using outer volume suppression pulses, better image quality for diffusion‐weighted ssEPI acquisition has been achieved.^49^, ^52^ Multishot or segmented EPI has been implemented as a substitute for ssEPI to overcome reduced image quality caused by susceptibility artifacts, which allows for improved spatial resolution. However, this technique elongates the acquisition time, which increases the risk of motion artifacts.

Fast spin‐echo (FSE) or turbo spin‐echo (TSE) sequences use spin‐echoes instead of gradient‐echoes, which renders them desirable against susceptibility variations and geometric distortions (Fig. 5). However, the maximum spatial resolution allowed by these sequences cannot surpass that of the ssEPI method, due to fast decay of T_2_ signal. The steady‐state free‐precession (SSFP) technique extended by diffusion gradient pulse has successfully been applied for imaging of bone marrow, although it is difficult to relate the measured signal to the diffusion coefficient.^49^, ^52^

In terms of signal modeling, the proportion of fat and water components within the bone marrow, as well as the abundance of perfusion provided for the tissue, is different in various anatomical regions. Diffusion measurements in bone depend on the imaging site, which causes implications for the choice of sequence parameters, including fat‐suppression and b‐values, and consequently affects the accuracy of estimations of diffusion parameters. The choice of b‐values for different bone sites can affect ADC measures, as restriction of water diffusion varies from lowest to highest in vertebrae through the femoral neck to calcaneus, resulting in ADC values ranging from lowest to highest in these locations. For example, fat comprises 50–70% of the vertebral bone marrow, while it forms 60–80% of femoral neck and 78–98% of the calcaneus of healthy postmenopausal women.^7^ Hence, the amount of interstitial space between bone and marrow fat, where diffusion of water occurs, varies depending on the anatomical location. Furthermore, the pore size also varies between these regions. At different locations of lumbar spine^104^, ^105^ and different ages of subjects,^57^, ^93^ diverse diffusion coefficients of normal vertebral bone marrow have been reported. These issues become more severe in studying osteoporotic patients: in these cases, competing factors of an increase in fat component and expansion of pore space occur, where the former decrements and the latter increments ADC values. Thus, b‐values should be tailored in correspondence with the desired ADC values within the tissue (with approximately a reverse relationship: *b* ≈ 1/*ADC*). There is a trade‐off between reaching a sufficient SNR of diffusion images and adequate weighting of diffusion signal to recognize slow diffusion of water within the bone marrow.^61^ With smaller b‐values (<100 mm^2^/s), the influence of perfusion produces biased higher ADC values. Larger b‐values (>600 mm^2^/s) are desired for imaging regions with lower ADCs, like femoral neck and calcaneus, but require longer scan times and have lower SNR. In several studies, b‐values are adjusted between 50 (to reduce perfusion effects) and 600 mm^2^/s for vertebral bone marrow imaging,^52^ 2500 mm^2^/s for femoral neck and 8000 mm^2^/s for calcaneus.^61^

Conventionally, chemical shift artifacts and contamination of water diffusion by fat signal are handled by applying fat‐suppression techniques, including spectral‐selective or combined spectral‐selective and inversion recovery methods.^52^ While these methods can suppress the main fat peaks (positioned between 0 and 3 ppm), the peak belonging to olefinic and glycerol fat near the water peak (4.7 ppm) cannot be suppressed. As the diffusion coefficient of bone marrow, water, and fat are distinctly dissimilar, this residual fat peak can bias the measurements of bone marrow ADC.^49^, ^103^

The IMFG effect is another relevant factor for selection of b‐values for diffusion imaging, also dependent on the anatomical site. The magnetic field gradient sensed by diffusing water molecules adjacent to the interface of marrow and solid bone becomes greater when this interface narrows, and if located in higher magnetic fields. This is because the diffusive motion of water in IMFGs produces nonreversible dephasing. Therefore, if the susceptibility difference between water and bone is not considered, the measured ADC would be different from the actual ADC.^7^

## Discussion

dMRI can be customized to acquire information from several aspects of tissue properties. In particular, in bone imaging, dMRI can potentially detect variations of bone marrow fat content, water content within cancellous bone, perfusion, anisotropic microarchitecture of cancellous bone, and fluid flow within cortical bone.

In many diseases, physiological changes precede structural variations and, therefore, the potential of dMRI in revealing perfusion and marrow fat and water contents is encouraging for devising tools for early diagnosis. For osteoporosis, early detection of bone tissue variations at the beginning or during disease progression might provide a means for identifying causes, early treatment, and a higher chance of maintaining the quality of life and life expectancy of patients.

A main problem with most of bone (especially bone marrow) diffusion studies arise from limitations in acquisition hardware and techniques, feasibility of acquiring multiple b‐values trading off scan time and SNR, and lack of a suitable multicomponent analysis method. For bone marrow, the compartments comprise water, fat, and perfusion, overall forming at least three coexisting components within the marrow. According to different studies, we expect the perfusion fraction (*f*) and fat fraction (FF) to decrease during aging and osteoporosis. Furthermore, they vary in association with anatomical region and gender, in a way that *f* and FF are expected to decrease from upper body to lower body sites, *f* to be higher in females than males before 50 years of age and decrease in females older than 50 years, and FF to be higher in females. When these components are isolated from the diffusion coefficient, considering the reduction of marrow cellularity during aging and osteoporosis, *D* is expected to decrease as well. Therefore, dMRI acquisition and modeling should take these variations of age, gender, and anatomical location of interest into account.

The majority of available dMRI research studies in bone ignore the effect of anisotropy of trabecular bone microarchitecture using an isotropic ADC instead. Most dMRI studies have focused on evaluating the mean diffusivity properties of bone marrow water components, with only a few preliminary in vivo studies quantifying FA in cancellous bone.^72^, ^73^ Only when combined with FF measured by MRS could these works find a relationship between DTI‐derived parameters (viz. MD and FA) and BMD variations. Even using fat‐suppression during DTI acquisition, considering the small size of the studied population, it is difficult to confirm from these results whether the residual olefinic/glycerol fat component played a role in producing a significant correlation between MD with MRS‐derived FF in healthy volunteers and between FA and FF in osteopenic/osteoporotic patients. This encourages the importance of considering multicomponent quantification besides multidirectional dMRI. A possible alternative to separately acquiring DWI and MRS, like the aforementioned studies,^72^, ^73^ could be emerging DW‐MRS techniques that integrate quantitative metrics from both modalities.^106^ Nonetheless, these methods are in their infancy stages and have not yet been tested clinically.

In terms of dMRI protocols, it is important to pay attention to the choice of sequences, as it can introduce artifacts if not correctly adjusted. With modifications of EPI and the introduction of ssEPI, segmented or reduced FOV EPI techniques, the challenges with bone marrow imaging have largely diminished. Numerous studies have removed the confounding effect of fat through applying fat‐suppression techniques. However, a component of fat that resides near water peak cannot be eliminated. Furthermore, quantification of changes in the marrow fat component provides a helpful biomarker for the diagnosis of osteoporosis in terms of marrow adiposity, implying the importance of choosing a more representative compartmental model for quantification of several components of marrow, instead of suppressing them. IMFG has been considered a challenge to be avoided in most dMRI studies. Nevertheless, through careful acquisition and quantification, it could serve as a beneficial effect that can help to visualize the microarchitecture of trabecular bone. Finally, the number of orientations for diffusion acquisition can be extended to more than the default three directions, to model the anisotropic diffusion of water. This approach is beneficial for trabecular bone imaging and especially in osteoporosis, as structural deformations can be detected along with physiological changes.

With current advances and according to the literature, optimizing pulse sequences to acquire multi‐b‐value and multidirectional dMRI in bone marrow imaging seems plausible in vivo and in clinical applications. Currently, quantification of dMRI in bone is solely based on the IVIM model. By extending dMRI quantification to multicompartmental models of bone, assessment of different contributing factors to bone aging and osteoporosis becomes feasible. DDIF has been used in a few clinical applications and, through extensive explorations on larger datasets, can become useful in assessing trabecular bone architecture in aging and osteoporosis. Additionally, while the diffusion process plays an important role in mechano‐transduction of bone, and bone water content changes is a helpful biomarker for diagnosing aging and osteoporosis, only one ex vivo study has explored the use of dMRI to examine cortical bone water content.^65^ The transverse relaxation decay of cortical bone water pools is very fast, while their diffusion coefficient is very small (*D* = 0.0078 × 10^‐3^ mm^2^/s)^65^; thus, it takes several minutes for transportation to take place in cortical bone. Furthermore, motion is a critical challenge in cortical bone water imaging and especially for implementation of a diffusion pulse sequence. Hence, with current hardware specifications, it is impossible to acquire dMRI in cortical bone in vivo. With the advent of new technological advances and with more ex vivo studies, this measurement may become feasible in vivo.

In conclusion, diffusion imaging in osteoporosis offers promising potential but it is technically challenging, particularly in establishing a compromise between imaging and modeling demands. Nonetheless, with proper imaging and the advent of new quantitative models, diffusion MRI offers valuable biomarkers for detection of multiple contributing elements to osteoporosis‐related bone tissue variations.

## Supporting information


**Appendix S1**: Supplementary Information

## References

[1] Lorentzon M , Cummings SR . Osteoporosis: The evolution of a diagnosis. J Intern Med 2015;277:650–661. 25832448 10.1111/joim.12369

[2] Griffith JF , Genant HK . Bone mass and architecture determination: State of the art. Best Pract Res Clin Endocrinol Metab 2008;22:737–764. 19028355 10.1016/j.beem.2008.07.003

[3] Wehrli FW . Structural and functional assessment of trabecular and cortical bone by micro magnetic resonance imaging. J Magn Reson Imaging 2007;25:390–409. 17260403 10.1002/jmri.20807

[4] Organization WH . Assessment of fracture risk and its application to screening for postmenopausal osteoporosis: Report of a WHO study group [meeting held in Rome from 22 to 25 June 1992] 1994. 7941614

[5] Griffith JF , Genant HK . New advances in imaging osteoporosis and its complications. Endocrine 2012;42:39–51. 22618377 10.1007/s12020-012-9691-2

[6] Wehrli FW , Song HK , Saha PK , Wright AC . Quantitative MRI for the assessment of bone structure and function. NMR Biomed 2006;19:731–764. 17075953 10.1002/nbm.1066

[7] Capuani S , Manenti G , Iundusi R , Tarantino U . Focus on diffusion MR investigations of musculoskeletal tissue to improve osteoporosis diagnosis: A brief practical review. BioMed Res Int 2015;2015. 10.1155/2015/948610 PMC4377366 25861652

[8] Eyre DR . Bone biomarkers as tools in osteoporosis management. Spine 1997;22:17S–24S. 9431640 10.1097/00007632-199712151-00004

[9] Robey PG , Boskey AL. The composition of bone. Primer on the bone metabolic diseases and disorders of mineral metabolism, 7th ed. Am Soc Bone Min Res 2009:32–38.

[10] Buckwalter J , Glimcher M , Cooper R , Recker R . Bone biology. Part I: Structure, blood supply, cells, matrix, and mineralization. JBJS 1995;77:1256–1275. 8727757

[11] Chang G , Boone S , Martel D , et al. MRI assessment of bone structure and microarchitecture. J Magn Reson Imaging 2017;46:323–337. 28165650 10.1002/jmri.25647 PMC5690546

[12] Manolagas SC , Jilka RL . Bone marrow, cytokines, and bone remodeling—Emerging insights into the pathophysiology of osteoporosis. N Engl J Med 1995;332:305–311. 7816067 10.1056/NEJM199502023320506

[13] Bermeo S , Gunaratnam K , Duque G . Fat and bone interactions. Curr Osteoporos Rep 2014;12:235–242. 24599601 10.1007/s11914-014-0199-y

[14] Wehrli FW , Fernández‐Seara MA . Nuclear magnetic resonance studies of bone water. Ann Biomed Eng 2005;33:79–86. 15709708 10.1007/s10439-005-8965-8

[15] Fernández‐Seara MA , Wehrli SL , Takahashi M , Wehrli FW . Water content measured by proton‐deuteron exchange NMR predicts bone mineral density and mechanical properties. J Bone Min Res 2004;19:289–296. 10.1359/JBMR.0301227 14969399

[16] Cowin SC , Gailani G , Benalla M . Hierarchical poroelasticity: Movement of interstitial fluid between porosity levels in bones. Philos Trans A Math Phys Eng Sci 2009;367:3401–3444. 19657006 10.1098/rsta.2009.0099

[17] Lemaire T , Pham TT , Capiez‐Lernout E , de Leeuw NH , Naili S. Water in hydroxyapatite nanopores: Possible implications for interstitial bone fluid flow. J Biomech 2015;48:3066–3071. 26283410 10.1016/j.jbiomech.2015.07.025

[18] Horch RA , Gochberg DF , Nyman JS , Does MD . Clinically compatible MRI strategies for discriminating bound and pore water in cortical bone. Magn Reson Med 2012;68:1774–1784. 22294340 10.1002/mrm.24186 PMC3357454

[19] Cowin S , Weinbaum S , Zeng Y . A case for bone canaliculi as the anatomical site of strain generated potentials. J Biomech 1995;28:1281–1297. 8522542 10.1016/0021-9290(95)00058-p

[20] Skedros JG , Hunt KJ , Bloebaum RD . Relationships of loading history and structural and material characteristics of bone: Development of the mule deer calcaneus. J Morphol 2004;259:281–307. 14994328 10.1002/jmor.10167

[21] Tanck E , Hannink G , Ruimerman R , Buma P , Burger EH , Huiskes R. Cortical bone development under the growth plate is regulated by mechanical load transfer. J Anat 2006;208:73–79. 16420380 10.1111/j.1469-7580.2006.00503.x PMC2100179

[22] Cowin SC , Cardoso L . Blood and interstitial flow in the hierarchical pore space architecture of bone tissue. J Biomech 2015;48:842–854. 25666410 10.1016/j.jbiomech.2014.12.013 PMC4489573

[23] Hemmatian H , Bakker AD , Klein‐Nulend J , van Lenthe GH . Aging, Osteocytes, and Mechanotransduction. Curr Osteoporos Rep 2017;15:401–411. 28891009 10.1007/s11914-017-0402-z PMC5599455

[24] Nyman JS , Ni Q , Nicolella DP , Wang X . Measurements of mobile and bound water by nuclear magnetic resonance correlate with mechanical properties of bone. Bone 2008;42:193–199. 17964874 10.1016/j.bone.2007.09.049 PMC2275807

[25] Li C , Seifert AC , Rad HS , et al. Cortical bone water concentration: Dependence of MR imaging measures on age and pore volume fraction. Radiology 2014;272:796–806. 24814179 10.1148/radiol.14132585 PMC4263649

[26] Granke M , Does MD , Nyman JS . The role of water compartments in the material properties of cortical bone. Calcif Tissue Int 2015;97:292–307. 25783011 10.1007/s00223-015-9977-5 PMC4526331

[27] Marenzana M , Arnett TR . The key role of the blood supply to bone. Bone Res 2013;1:203–215. 26273504 10.4248/BR201303001 PMC4472103

[28] Sivaraj KK , Adams RH . Blood vessel formation and function in bone. Development 2016;143:2706–2715. 27486231 10.1242/dev.136861

[29] Spier SA , Delp MD , Meininger CJ , Donato AJ , Ramsey MW , Muller‐Delp JM . Effects of ageing and exercise training on endothelium‐dependent vasodilatation and structure of rat skeletal muscle arterioles. The Journal of physiology 2004;556:947–958. 15004211 10.1113/jphysiol.2003.060301 PMC1665008

[30] Chim SM , Tickner J , Chow ST , et al. Angiogenic factors in bone local environment. Cytokine & growth factor reviews 2013;24:297–310. 23611723 10.1016/j.cytogfr.2013.03.008

[31] Losordo DW , Isner JM . Estrogen and angiogenesis: A review. Arteriosclerosis, thrombosis, and vascular biology 2001;21:6–12. 10.1161/01.atv.21.1.6 11145928

[32] Laroche M . Intraosseous circulation from physiology to disease. Joint Bone Spine 2002;69:262–269. 12102272 10.1016/s1297-319x(02)00391-3

[33] Griffith JF , Wang YX , Zhou H , et al. Reduced bone perfusion in osteoporosis: Likely causes in an ovariectomy rat model. Radiology 2010;254:739–746. 20177089 10.1148/radiol.09090608

[34] Griffith JF , Yeung DK , Tsang PH , et al. Compromised bone marrow perfusion in osteoporosis. J Bone Min Res 2008;23:1068–1075. 10.1359/jbmr.080233 18302498

[35] Dinenno FA , Tanaka H , Stauffer BL , Seals DR . Reductions in basal limb blood flow and vascular conductance with human ageing: Role for augmented α‐adrenergic vasoconstriction. J Physiol 2001;536:977–983. 11691889 10.1111/j.1469-7793.2001.00977.x PMC2278891

[36] Ma HT , Griffith JF , Zhao X , Lv H , Yeung DK , Leung P‐C . Relationship between marrow perfusion and bone mineral density: A pharmacokinetic study of DCE‐MRI. Engineering in Medicine and Biology Society (EMBC), 2012 Annual International Conference of the IEEE; 2012 p 377–379. 10.1109/EMBC.2012.6345947 23365908

[37] Dyke JP , Lazaro LE , Hettrich CM , Hentel KD , Helfet DL , Lorich DG . Regional analysis of femoral head perfusion following displaced fractures of the femoral neck. J Magn Reson Imaging 2015;41:550–554. 24338938 10.1002/jmri.24524

[38] Wang YX , Griffith JF , Kwok AW , et al. Reduced bone perfusion in proximal femur of subjects with decreased bone mineral density preferentially affects the femoral neck. Bone 2009;45:711–715. 19555783 10.1016/j.bone.2009.06.016

[39] Griffith JF , Yeung DK , Ma HT , Leung JC , Kwok TC , Leung PC . Bone marrow fat content in the elderly: A reversal of sex difference seen in younger subjects. J Magn Reson Imaging 2012;36:225–230. 22337076 10.1002/jmri.23619

[40] Berg BCV , Omoumi P , Galant C , Michoux N , Lecouvet F . MR imaging of the normal bone marrow and normal variants. Magnetic resonance imaging of the bone marrow. Berlin: Springer; 2013 p 21–46.

[41] Bartl R . Histology of normal bone and bone marrow, and their main disorders. Magnetic resonance imaging of the bone marrow. Berlin: Springer; 2013 p 3–20.

[42] Budzik JF , Lefebvre G , Forzy G , El Rafei M , Chechin D , Cotten A . Study of proximal femoral bone perfusion with 3D T1 dynamic contrast‐enhanced MRI: A feasibility study. Eur Radiol 2014;24:3217–3223. 25120203 10.1007/s00330-014-3340-5

[43] Schwartz AV . Marrow fat and bone: Review of clinical findings. Front Endocrinol 2015;6:40. 10.3389/fendo.2015.00040 PMC4378315 25870585

[44] Cordes C , Baum T , Dieckmeyer M , et al. MR–based assessment of bone marrow fat in osteoporosis, diabetes, and obesity. Front Endocrinol 2016;7:74. 10.3389/fendo.2016.00074 PMC4921741 27445977

[45] Rosen CJ , Ackert‐Bicknell C , Rodriguez JP , Pino AM . Marrow fat and the bone microenvironment: Developmental, functional, and pathological implications. Crit Rev Eukaryotic Gene Expr 2009;19(2). 10.1615/critreveukargeneexpr.v19.i2.20 PMC2674609 19392647

[46] Hardouin P , Pansini V , Cortet B . Bone marrow fat. Joint Bone Spine 2014;81:313–319. 24703396 10.1016/j.jbspin.2014.02.013

[47] Kugel H , Jung C , Schulte O , Heindel W . Age‐ and sex‐specific differences in the 1H‐spectrum of vertebral bone marrow. J Magn Reson Imaging 2001;13:263–268. 11169833 10.1002/1522-2586(200102)13:2<263::aid-jmri1038>3.0.co;2-m

[48] Li X , Kuo D , Schafer AL , et al. Quantification of vertebral bone marrow fat content using 3 Tesla MR spectroscopy: Reproducibility, vertebral variation, and applications in osteoporosis. J Magn Reson Imaging 2011;33:974–979. 21448966 10.1002/jmri.22489 PMC3072841

[49] Karampinos DC , Ruschke S , Dieckmeyer M , et al. Quantitative MRI and spectroscopy of bone marrow. J Magn Reson Imaging 2018;47:332–353. 28570033 10.1002/jmri.25769 PMC5811907

[50] Rad HS , Lam SCB , Magland JF , et al. Quantifying cortical bone water in vivo by three‐dimensional ultra‐short echo‐time MRI. NMR Biomed 2011;24:855–864. 21274960 10.1002/nbm.1631 PMC3684973

[51] Techawiboonwong A , Song HK , Wehrli FW . In vivo MRI of submillisecond T2 species with two‐dimensional and three‐dimensional radial sequences and applications to the measurement of cortical bone water. NMR Biomed 2008;21:59–70. 17506113 10.1002/nbm.1179

[52] Dietrich O , Geith T , Reiser MF , Baur‐Melnyk A . Diffusion imaging of the vertebral bone marrow. NMR Biomed 2017;30(3). 10.1002/nbm.3333 26114411

[53] Jones DK . Studying connections in the living human brain with diffusion MRI. Cortex 2008;44:936–952. 18635164 10.1016/j.cortex.2008.05.002

[54] Hagmann P , Jonasson L , Maeder P , Thiran J‐P , Wedeen VJ , Meuli R . Understanding diffusion MR imaging techniques: From scalar diffusion‐weighted imaging to diffusion tensor imaging and beyond. Radiographics 2006;26(suppl_1):S205–S223. 17050517 10.1148/rg.26si065510

[55] Biffar A , Dietrich O , Sourbron S , Duerr HR , Reiser MF , Baur‐Melnyk A . Diffusion and perfusion imaging of bone marrow. Eur J Radiol 2010;76:323–328. 20381277 10.1016/j.ejrad.2010.03.011

[56] Wehrli FW . Magnetic resonance of calcified tissues. J Magn Reson 2013;229:35–48. 23414678 10.1016/j.jmr.2012.12.011 PMC4746726

[57] Herrmann J , Krstin N , Schoennagel BP , et al. Age‐related distribution of vertebral bone‐marrow diffusivity. Eur J Radiol 2012;81:4046–4049. 23017195 10.1016/j.ejrad.2012.03.033

[58] Nonomura Y , Yasumoto M , Yoshimura R , et al. Relationship between bone marrow cellularity and apparent diffusion coefficient. J Magn Reson Imaging 2001;13:757–760. 11329198 10.1002/jmri.1105

[59] Jaramillo D , Connolly SA , Vajapeyam S , et al. Normal and ischemic epiphysis of the femur: Diffusion MR imaging—Study in piglets. Radiology 2003;227:825–832. 12773684 10.1148/radiol.2273011673

[60] Li Q , Pan S‐n , Yin Y‐m , et al. Normal cranial bone marrow MR imaging pattern with age‐related ADC value distribution. Eur J Radiol 2011;80:471–477. 20950974 10.1016/j.ejrad.2010.09.010

[61] Capuani S . Water diffusion in cancellous bone. Micropor Mesopor Mater 2013;178:34–38.

[62] Lavdas I , Rockall AG , Castelli F , et al. Apparent diffusion coefficient of normal abdominal organs and bone marrow from whole‐body DWI at 1.5 T: The effect of sex and age. AJR Am J Roentgenol 2015;205:242–250. 26204271 10.2214/AJR.14.13964

[63] He J , Fang H , Na Li X . Vertebral bone marrow diffusivity in normal adults with varying bone densities at 3T diffusion‐weighted imaging. Acta Radiol 2017:284185117704235. 10.1177/0284185117704235 28409528

[64] Griffith JF , Yeung DK , Antonio GE , et al. Vertebral marrow fat content and diffusion and perfusion indexes in women with varying bone density: MR evaluation. Radiology 2006;241:831–838. 17053202 10.1148/radiol.2413051858

[65] Fernandez‐Seara MA , Wehrli SL , Wehrli FW . Diffusion of exchangeable water in cortical bone studied by nuclear magnetic resonance. Biophys J 2002;82(1 Pt 1):522–529. 11751339 10.1016/S0006-3495(02)75417-9 PMC1302492

[66] Beaulieu C. The biological basis of diffusion anisotropy. Diffusion MRI, 2nd ed Amsterdam: Elsevier; 2014 p 155–183.

[67] Iima M , Le Bihan D . Clinical intravoxel incoherent motion and diffusion MR imaging: past, present, and future. Radiology 2016;278:13–32. 26690990 10.1148/radiol.2015150244

[68] Capuani S , Rossi C , Alesiani M , Maraviglia B. Diffusion tensor imaging to study anisotropy in a particular porous system: The trabecular bone network. Solid State Nucl Magn Reson 2005;28:266–272. 16360582 10.1016/j.ssnmr.2005.11.001

[69] Jones DK . Gaussian modeling of the diffusion signal. Diffusion MRI, 2nd ed Amsterdam: Elsevier; 2014 p 87–104.

[70] Johansen‐Berg H , Behrens TE. Diffusion MRI : From quantitative measurement to in vivo neuroanatomy. New York: Academic Press; 2013.

[71] Rossi C , Capuani S , Fasano F , Alesiani M , Maraviglia B . DTI of trabecular bone marrow. Magn Reson Imaging 2005;23:245–248. 15833620 10.1016/j.mri.2004.11.018

[72] Manenti G , Capuani S , Fanucci E , et al. Diffusion tensor imaging and magnetic resonance spectroscopy assessment of cancellous bone quality in femoral neck of healthy, osteopenic and osteoporotic subjects at 3T: Preliminary experience. Bone 2013;55:7–15. 23507402 10.1016/j.bone.2013.03.004

[73] Manenti G , Capuani S , Fusco A , Fanucci E , Tarantino U , Simonetti G . Osteoporosis detection by 3T diffusion tensor imaging and MRI spectroscopy in women older than 60 years. Aging Clin Exp Res 2013;25(Suppl 1):S31–34. 24046053 10.1007/s40520-013-0091-0

[74] Bouchard LS , Wehrli FW , Chin CL , Warren WS . Structural anisotropy and internal magnetic fields in trabecular bone: Coupling solution and solid dipolar interactions. J Magn Reson 2005;176:27–36. 15953742 10.1016/j.jmr.2005.05.012

[75] Ma J , Wehrli FW . Method for image‐based measurement of the reversible and irreversible contribution to the transverse‐relaxation rate. J Magn Reson B 1996;111:61–69. 8620286 10.1006/jmrb.1996.0060

[76] Chung H , Wehrli F , Williams J , Kugelmass S . Relationship between NMR transverse relaxation, trabecular bone architecture, and strength. Proc Natl Acad Sci U S A 1993;90:10250–10254. 8234285 10.1073/pnas.90.21.10250 PMC47752

[77] Majumdar S , Thomasson D , Shimakawa A , Genant H . Quantitation of the susceptibility difference between trabecular bone and bone marrow: Experimental studies. Magn Reson Med 1991;22:111–127. 1798386 10.1002/mrm.1910220112

[78] Hwang SN , Wehrli FW . The calculation of the susceptibility‐induced magnetic field from 3D NMR images with applications to trabecular bone. J Magn Reson B 1995;109:126–145.

[79] Song YQ . Using internal magnetic fields to obtain pore size distributions of porous media. Concepts Magn Reson A 2003;18:97–110.

[80] Lisitza N , Song Y‐Q . The behavior of diffusion eigenmodes in the presence of internal magnetic field in porous media. J Chem Phys 2001;114:9120–9124.

[81] Sigmund E , Cho H , Chen P , et al. Diffusion‐based MR methods for bone structure and evolution. Magn Reson Med 2008;59:28–39. 18098292 10.1002/mrm.21281

[82] Sigmund E , Cho H , Song YQ . High‐resolution MRI of internal field diffusion‐weighting in trabecular bone. NMR Biomed 2009;22:436–448. 19023866 10.1002/nbm.1354

[83] Sprinkhuizen SM , Ackerman JL , Song YQ . Influence of bone marrow composition on measurements of trabecular microstructure using decay due to diffusion in the internal field MRI: Simulations and clinical studies. Magn Reson Med 2014;72:1499–1508. 24382681 10.1002/mrm.25061 PMC4077997

[84] Rebuzzi M , Vinicola V , Taggi F , Sabatini U , Wehrli FW , Capuani S . Potential diagnostic role of the MRI‐derived internal magnetic field gradient in calcaneus cancellous bone for evaluating postmenopausal osteoporosis at 3T. Bone 2013;57:155–163. 23899635 10.1016/j.bone.2013.07.027

[85] De Santis S , Rebuzzi M , Di Pietro G , Fasano F , Maraviglia B , Capuani S. In vitro and in vivo MR evaluation of internal gradient to assess trabecular bone density. Phys Med Biol 2010;55:5767–5785. 20844335 10.1088/0031-9155/55/19/010

[86] Álvarez GA , Shemesh N , Frydman L . Internal gradient distributions: A susceptibility‐derived tensor delivering morphologies by magnetic resonance. Sci Rep 2017;7:3311. 28607445 10.1038/s41598-017-03277-9 PMC5468317

[87] Le Bihan D , Iima M . Diffusion magnetic resonance imaging: What water tells us about biological tissues. PLoS Biol 2015;13:e1002203. 10.1371/journal.pbio.1002246 PMC4559450 26334873

[88] Le Bihan D . Intravoxel incoherent motion perfusion MR imaging: A wake‐up call. Radiology 2008;249:748–752. 19011179 10.1148/radiol.2493081301

[89] Le Bihan D . Intravoxel incoherent motion imaging using steady‐state free precession. Magn Reson Med 1988;7:346–351. 3205150 10.1002/mrm.1910070312

[90] Le Bihan D , Breton E , Lallemand D , Aubin M , Vignaud J , Laval‐Jeantet M . Separation of diffusion and perfusion in intravoxel incoherent motion MR imaging. Radiology 1988;168:497–505. 3393671 10.1148/radiology.168.2.3393671

[91] Le Bihan D . What can we see with IVIM MRI? NeuroImage 2019;187:56–67. 29277647 10.1016/j.neuroimage.2017.12.062

[92] Shah R , Stieltjes B , Andrulis M , et al. Intravoxel incoherent motion imaging for assessment of bone marrow infiltration of monoclonal plasma cell diseases. Ann Hematol 2013;92:1553–1557. 23680869 10.1007/s00277-013-1786-1

[93] Yeung DK , Wong SY , Griffith JF , Lau EM . Bone marrow diffusion in osteoporosis: Evaluation with quantitative MR diffusion imaging. J Magn Reson Imaging 2004;19:222–228. 14745757 10.1002/jmri.10453

[94] Marchand AJ , Hitti E , Monge F , et al. MRI quantification of diffusion and perfusion in bone marrow by intravoxel incoherent motion (IVIM) and non‐negative least square (NNLS) analysis. Magn Reson Imaging 2014;32:1091–1096. 25093628 10.1016/j.mri.2014.07.009

[95] Bourillon C , Rahmouni A , Lin C , et al. Intravoxel incoherent motion diffusion‐weighted imaging of multiple myeloma lesions: Correlation with whole‐body dynamic contrast agent‐enhanced MR imaging. Radiology 2015;277:773–783. 26131910 10.1148/radiol.2015141728

[96] Baik JS , Jung JY , Jee WH , et al. Differentiation of focal indeterminate marrow abnormalities with multiparametric MRI. J Magn Reson Imaging 2017;46:49–60. 27859835 10.1002/jmri.25536

[97] Lee EYP , Perucho JAU , Vardhanabhuti V , et al. Intravoxel incoherent motion MRI assessment of chemoradiation‐induced pelvic bone marrow changes in cervical cancer and correlation with hematological toxicity. J Magn Reson Imaging 2017;46:1491–1498. 28225579 10.1002/jmri.25680

[98] Niu J , Li W , Wang H , et al. Intravoxel incoherent motion diffusion‐weighted imaging of bone marrow in patients with acute myeloid leukemia: A pilot study of prognostic value. J Magn Reson Imaging 2017;46:476–482. 28211619 10.1002/jmri.25600

[99] Park S , Kwack K‐S , Chung N‐S , Hwang J , Lee HY , Kim JH . Intravoxel incoherent motion diffusion‐weighted magnetic resonance imaging of focal vertebral bone marrow lesions: Initial experience of the differentiation of nodular hyperplastic hematopoietic bone marrow from malignant lesions. Skeletal Radiol 2017;46:675–683. 28265697 10.1007/s00256-017-2603-z

[100] Yoon MA , Hong S‐J , Lee CH , Kang CH , Ahn K‐S , Kim BH . Intravoxel incoherent motion (IVIM) analysis of vertebral bone marrow changes after radiation exposure from diagnostic imaging and interventional procedures. Acta Radiol 2017;58:1260–1268. 28103708 10.1177/0284185116688380

[101] Ohno N , Miyati T , Kasai H , et al. Evaluation of perfusion‐related and true diffusion in vertebral bone marrow: A preliminary study. Radiol Phys Technol 2015;8:135–140. 25413477 10.1007/s12194-014-0301-2

[102] Biffar A , Baur‐Melnyk A , Schmidt GP , Reiser MF , Dietrich O . Quantitative analysis of the diffusion‐weighted steady‐state free precession signal in vertebral bone marrow lesions. Invest Radiol 2011;46:601–609. 21610504 10.1097/RLI.0b013e31821e637d

[103] Dieckmeyer M , Ruschke S , Eggers H , et al. ADC quantification of the vertebral bone marrow water component: Removing the confounding effect of residual fat. Magn Reson Med 2017;78:1432–1441. 27851874 10.1002/mrm.26550

[104] Hillengass J , Stieltjes B , Bäuerle T , et al. Dynamic contrast‐enhanced magnetic resonance imaging (DCE‐MRI) and diffusion‐weighted imaging of bone marrow in healthy individuals. Acta Radiol 2011;52:324–330. 21498370 10.1258/ar.2010.100366

[105] Eguchi Y , Ohtori S , Yamashita M , et al. Diffusion magnetic resonance imaging to differentiate degenerative from infectious endplate abnormalities in the lumbar spine. Spine 2011;36:E198–E202. 21099738 10.1097/BRS.0b013e3181d5ff05

[106] Ruschke S , Kienberger H , Baum T , et al. Diffusion‐weighted stimulated echo acquisition mode (DW‐STEAM) MR spectroscopy to measure fat unsaturation in regions with low proton‐density fat fraction. Magn Reson Med 2016;75:32–41. 25753506 10.1002/mrm.25578

[107] Tomlinson RE , Silva MJ . Skeletal blood flow in bone repair and maintenance. Bone Res 2013;1:311–322. 26273509 10.4248/BR201304002 PMC4472118

[108] Dyke JP , Aaron RK . Noninvasive methods of measuring bone blood perfusion. Ann N Y Acad Sci 2010;1192:95–102. 20392223 10.1111/j.1749-6632.2009.05376.x PMC2894463

[109] Ou‐Yang L , Lu GM . Dysfunctional microcirculation of the lumbar vertebral marrow prior to the bone loss and intervertebral discal degeneration. Spine 2015;40:E593–600. 25955095 10.1097/BRS.0000000000000834 PMC4431500

[110] Ou‐Yang L , Lu GM . Decrease with aging of the microcirculatory function of the lumbar vertebral marrow preceding the loss of bone material density and the onset of intervertebral discal degeneration: A study about the potential cause. Chron Dis Transl Med 2015;1:96–104. 10.1016/j.cdtm.2015.02.007 PMC5643569 29062993

[111] Ma HT , Griffth JF , Leung PC . Muscle‐based pharmacokinetic modeling of marrow perfusion for osteoporotic bone in females. Biomed Res Int 2014;2014:620925. 25003121 10.1155/2014/620925 PMC4070517

[112] Ma HT , Griffith JF , Yeung DK , Leung PC . Modified brix model analysis of bone perfusion in subjects of varying bone mineral density. J Magn Reson Imaging 2010;31:1169–1175. 20432353 10.1002/jmri.22164

[113] Griffith JF , Yeung DK , Antonio GE , et al. Vertebral bone mineral density, marrow perfusion, and fat content in healthy men and men with osteoporosis: Dynamic contrast‐enhanced MR imaging and MR spectroscopy. Radiology 2005;236:945–951. 16055699 10.1148/radiol.2363041425

[114] Griffith JF , Yeung DK , Chow SK , Leung JC , Leung PC . Reproducibility of MR perfusion and (1)H spectroscopy of bone marrow. J Magn Reson Imaging 2009;29:1438–1442. 19472419 10.1002/jmri.21765

[115] Griffith JF , Yeung DK , Leung JC , Kwok TC , Leung PC . Prediction of bone loss in elderly female subjects by MR perfusion imaging and spectroscopy. Eur Radiol 2011;21:1160–1169. 21225266 10.1007/s00330-010-2054-6

[116] Chen WT , Shih TT , Chen RC , et al. Blood perfusion of vertebral lesions evaluated with gadolinium‐enhanced dynamic MRI: In comparison with compression fracture and metastasis. J Magn Reson Imaging 2002;15:308–314. 11891976 10.1002/jmri.10063

[117] Chen WT , Shih TT , Chen RC , et al. Vertebral bone marrow perfusion evaluated with dynamic contrast‐enhanced MR imaging: Significance of aging and sex. Radiology 2001;220:213–218. 11426000 10.1148/radiology.220.1.r01jl32213

[118] Biffar A , Schmidt GP , Sourbron S , et al. Quantitative analysis of vertebral bone marrow perfusion using dynamic contrast‐enhanced MRI: Initial results in osteoporotic patients with acute vertebral fracture. J Magn Reson Imaging 2011;33:676–683. 21563252 10.1002/jmri.22497

[119] Wang YXJ , Zhang YF , Griffith JF , et al. Vertebral blood perfusion reduction associated with vertebral bone mineral density reduction: A dynamic contrast‐enhanced MRI study in a rat orchiectomy model. J Magn Reson Imaging 2008;28:1515–1518. 19025958 10.1002/jmri.21539

