# Supplementary material for: Diffusion MRI for Assessment of Bone Quality; A Review of Findings in Healthy Aging and Osteoporosis
Source: J Magn Reson Imaging. 2019 Nov 11;51(4):975–92. doi: 10.1002/jmri.26973 (PMC7078977; doi:10.1002/jmri.26973)
Supplement: Supplementary file 1 — Appendix S1: Supplementary Information [file JMRI-51-975-s001.docx]

**Manuscript ID:** JMRI-19-0711.R1

**Title:** Diffusion MRI For Assessment of Bone Quality; A Review of Findings in Healthy Aging and Osteoporosis

**Author(s):** Anahita Fathi Kazerooni, Jose M Pozo, Eugene Vincent McCloskey, Hamidreza Saligheh Rad, Alejandro F. Frangi

**Educational Objective**

Participant learning objectives must be stated in observable and measurable terms, and must be attainable within the time allotted to the program. Each objective should include a verb that is chosen to describe something a physician will do in practice (and not what the teacher will teach). Please ensure that the objective clearly states a standard against which one can judge the success in achieving the objective.

| **Upon completion of this educational activity, participants will be better able to:** |
| --- |
| objective 1: Describe the key factors contributing to variations in diffusion process in bone during healthy aging and osteoporosis |
| objective 2: Describe the potential role of diffusion MRI for assessment of bone quality in healthy aging and osteoporosis |

**Multiple Choice Questions**

Blackwell Futura Media Services adheres to similar question writing policies of the American Board of Internal Medicine (ABIM), [available here for reference](http://www.abim.org/about/examInfo/developed.aspx).

Each journal based activity contains 4 multiple choice questions (single best answer) that highlight the most important areas of the article, particularly those that highlight an impact on general practice.

- The questions should focus on what the physician is expected to know after reading the article without access to additional medical resources or references
- Incorrect answers should be designed to reflect plausible options
- The correct answer must be uncontroversial, evidence-based, and a better choice than the incorrect answers.

| # | Correct Answer | Questions/Answers | |
| --- | --- | --- | --- |
| 1. | B | What aspect of osteoporotic bone **cannot** be assessed *in-vivo* using current diffusion MRI techniques? | |
|  |  | A. | Bone marrow fat content |
|  |  | B. | Bound water pool |
|  |  | C. | Bone perfusion |
|  |  | D. | Structural anisotropy |
| 2. | A | According to the current studies on diffusion MRI in bone, *ADC*-value measurement significantly differs by all of the following **except**? | |
|  |  | A. | Osteopenia vs osteoporosis |
|  |  | B. | Anatomical location |
|  |  | C. | Patient’s gender |
|  |  | D. | Patient’s age |
| 3. | A | Which of the following statements regarding diffusion MRI in bone is **correct**? | |
|  |  | A. | Internal magnetic field gradients can provide helpful information for assessment of bone. |
|  |  | B. | Chemical shift artefacts can be handled using fat-suppression techniques. |
|  |  | C. | Spin-echo sequences surpass gradient-echo methods, in terms of maximum allowable spatial resolution. |
|  |  | D. | In osteoporotic patients, with enlargement of pores and increase in fat content *ADC* values decrease. |
| 4. | D | Which factor is relevant in selection of b-values in diffusion MR imaging of bone? | |
|  |  | A. | Scan time |
|  |  | B. | Signal to noise ratio (SNR) |
|  |  | C. | Interstitial space between bone and marrow fat |
|  |  | D. | All of the above are relevant |
